# Methylation profiling reveals novel molecular classes of rhabdomyosarcoma

**DOI:** 10.1038/s41598-021-01649-w

**Published:** 2021-11-15

**Authors:** Michael R. Clay, Anand Patel, Quynh Tran, Dale J. Hedges, Ti-Cheng Chang, Elizabeth Stewart, Greg Charville, Cynthia Cline, Michael A. Dyer, Brent A. Orr

**Affiliations:** 1 Department of Pathology, University of Colorado Hospital, Anschutz Inpatient Pavilion 1, 12605 East 16th Avenue, Room 3.003, Aurora, CO 80045 USA; 2 Department of Oncology, St. Jude Children’s Research Hospital, Memphis, USA; 3 Department of Pathology, St. Jude Children’s Research Hospital, 262 Danny Thomas Place, Memphis, TN MS25038105 USA; 4 Center for Applied Bioinformatics, St. Jude Children’s Research Hospital, Memphis, USA; 5 Department of Developmental Neurobiology, St. Jude Children’s Research Hospital, Memphis, USA; 6 Department of Pathology, Stanford Hospital and Clinics, Palo Alto, USA

**Keywords:** Sarcoma, Cancer epigenetics

## Abstract

Rhabdomyosarcomas (RMS) represent a family of aggressive soft tissue sarcomas that present in both children and adults. Pathologic risk stratification for RMS has been based on histologic subtype, with poor outcomes observed in alveolar rhabdomyosarcoma (ARMS) and the adult-type pleomorphic rhabdomyosarcoma (PRMS) compared to embryonal rhabdomyosarcoma (ERMS). Genomic sequencing studies have expanded the spectrum of RMS, with several new molecularly defined entities, including fusion-driven spindle cell/sclerosing rhabdomyosarcoma (SC/SRMS) and MYOD1-mutant SC/SRMS. Comprehensive genomic analysis has previously defined the mutational and copy number spectrum for the more common ERMS and ARMS and revealed corresponding methylation signatures. Comparatively, less is known about epigenetic correlates for the rare SC/SRMS or PRMS histologic subtypes. Herein, we present exome and RNA sequencing, copy number analysis, and methylation profiling of the largest cohort of molecularly characterized RMS samples to date. In addition to ARMS and ERMS, we identify two novel methylation subtypes, one having SC/SRMS histology and defined by MYOD1 p. L122R mutations and the other matching adult-type PRMS. Selected tumors from adolescent patients grouped with the PRMS methylation class, expanding the age range of these rare tumors. Limited follow-up data suggest that pediatric tumors with MYOD1-mutations are associated with an aggressive clinical course.

## Introduction

Rhabdomyosarcomas (RMS) are a family of aggressive soft tissue sarcomas presenting primarily in the pediatric population and more rarely in adults^1^. RMS are separated into distinct histologic variants, including pleomorphic rhabdomyosarcoma (PRMS), alveolar rhabdomyosarcoma (ARMS), embryonal rhabdomyosarcoma (ERMS), and the evolving category of spindle cell/sclerosing rhabdomyosarcoma (SC/SRMS)^1^. The two primary subtypes of RMS encountered in the pediatric population, ERMS and ARMS, can be distinguished molecularly as nearly 85% of ARMS are defined by gene fusions between *PAX3-FOXO1* or *PAX7-FOXO1*^1^*,* whereas ERMS are characterized by disparate mutations in the RAS pathway, effectors of the PI3 Kinase pathway, or in genes that control the cell cycle^2^. The spindle cell/sclerosing category of RMS is characterized by disparate presentations and recurrent molecular alterations, including *MYOD1*-mutant SC/SRMS^3^, interosseous SC/SRMS with *TFCP2* or *NCOA2* rearrangements^4^, and congenital SC/SRMS with *VGLL2, NCOA2,* or *CITED2* gene rearrangements^5,6^. Diagnosis of the *MYOD1*-mutant subtype of SC/SRMS, characterized by recurrent *MYOD1* p.L122R missense mutations, has important clinical implications as it is characterized by more aggressive clinical behavior^3,7^. PRMS, which primarily present in adults, is characterized by a complex karyotype and an absence of recurrent molecular alterations^8^.

Placing rhabdomyosarcoma into a specific pathologic subtype presents a significant challenge to clinical practice. Whereas a subset of morphologically ambiguous cases can be molecularly classified using *FOXO1* fusion status, fifteen percent of ARMS are fusion negative^9^. Similarly, SC/SRMS encompasses a variety of molecularly distinct entities that^1^ are exceptionally rare^2^, share morphologic overlap with ERMS, and^3^ are characterized by disparate molecular alterations for which testing is not routinely available in most pathology laboratories. In patients presenting in late adolescence or early adulthood, the distinction between pediatric ERMS with anaplasia and adult-type PRMS can be difficult, given the paucity of defining diagnostic molecular abnormalities in each group.

Several investigators have reported that RMS can be separated into at least two primary epigenetic groups based on differences in their genome-wide methylation profiles. These groups correspond closely to the embryonal and alveolar subtypes, with the latter being highly enriched for *PAX3-FOXO1* and *PAX7-FOXO1* fusions^10–12^. Despite these observations, complete representation of all subtypes of rhabdomyosarcoma has not been included in previous cohorts, leaving the question of how histologically-defined SC/SRMS and PRMS are epigenetically related to ERMS and ARMS.

We hypothesized that with sufficient representation, additional molecular subtypes could be identified in RMS. To test this hypothesis, we performed genome-wide methylation profiling on 154 rhabdomyosarcomas and unrelated pediatric skeletal muscle controls representing all primary histologic types of both adult and pediatric disease and correlated the findings with copy number profiling, next-generation sequencing, and clinical outcome analysis.

## Materials and methods

All methods were carried out in accordance with relevant guidelines and regulations. All experimental protocols were approved by the St. Jude Children’s Research Hospital Institutional Review Board (#XPD17-163). Informed consent was not required under the Office for Human Research Protections (OHRP) guidelines regarding the disposition of deidentified human tissues for human subjects research, and was waived by the St. Jude Children’s Research Hospital Institutional Review Board.

### Data generation and methylation array processing

One hundred and fifty-eight samples from St. Jude Children’s Research Hospital (SJCRH, pediatric) and Stanford Hospital and Clinics (adult) were analyzed using Illumina Methylation BeadChip (EPIC) arrays according to the manufacturer’s instructions. Data were generated from formalin-fixed paraffin-embedded (FFPE) tissue samples. DNA of 158 patients was extracted and hybridized to Illumina Infinium HumanMethylationEPIC BeadChip (850 K) arrays. All methylation data were analyzed in R (http://www.r-project.org, version 3.5.3), using multiple of packages from Bioconductor and other repositories. Specifically, array data were preprocessed using the *minfi* package (v.1.28.4)^13^. Background correction with dye-bias normalization was performed for all samples using noob (normal-exponential out-of-band) with the “single” dye method^14^. Filtering was performed to remove probes located on sex chromosomes, probes containing nucleotide polymorphisms (dbSNP132 Common) within five base pairs of the targeted CpG-site, or probes mapping to multiple sites on hg19 (allowing for one mismatch), and cross-reactive probes.

Survival analysis was performed after a manual chart review to extract survival status For deceased patients, the time from the initial diagnosis to death was calculated, and for live patients, the time from the initial diagnosis to the last clinic visit was calculated. Survival curves were generated using Graphpad Prism 9ing.

### Unsupervised clustering and copy number variation analysis

Principal component analysis was performed using the top 10,000 variably methylated probes. The number of statistically significant principal components was determined by agDimension function in the *PCDimension* package (v.1.1.11)^15^. Dimensionality reduction using 5 statistically significant principal components (*k* = 5) was used for Uniform Manifold Approximation and Projection (UMAP v.0.2.6)^16^ with non-default parameters: theta = 0, pca = F, perplexity = 4. To identify distinct clusters in the methylation data, the density-based spatial clustering of applications with noise (DBSCAN) algorithm^17^ was applied to the UMAP coordinates with *minPts* = 4 and *eps* = 0.65. To evaluate the separation of DBSCAN clusters in the UMAP coordinates, silhouette analysis was performed on the Euclidian distances among the samples using the R package *cluster* (v.2.1.0). For hierarchical clustering, Kendall correlation was calculated as a distance measure between samples using the top 10,000 most variable probes among the SJ samples, and the unsupervised hierarchical clustering was performed on the computed distance by complete linkage agglomeration method.

Copy number variation (CNV) analysis from methylation array data was performed using the *conumee* package (version 1.16.0)^18^. Chromosomal gain or loss was determined using a 0.18 threshold. Statistically significant frequent copy number variations (CNVs) were determined using GISTIC version 2.0.23^29^. Copy number profiles output as segments obtained from *conumee* R package were used as inputs for GISTIC2. Gain and loss were categorized with CNV values greater than 0.18 or smaller than -0.18, respectively. CNVs were also divided into those that are broad (defined as exceeding half of the length of a chromosome arm) and focal (shorter than this). We considered events with False Discovery Rate *q*-values < 0.25 as significant at a 90% confidence level. An “arm-level peel-off” correction was enabled to assign all CNVs in the same chromosome arm of the same sample to be part of a single event when determining whether multiple significantly recurrent events exist on that chromosome arm.

### Somatic mutation detection of matched tumor-normal WES samples

Paired-end sequencing reads were mapped with BWA^19^ to human genome GRCh38. We used an ensemble approach to call somatic mutations (SNV/indels) with multiple published tools, including Mutect2^20^, SomaticSniper^21^, VarScan2^22^, MuSE^23^, and Strelka2^24^. The consensus calls by at least two callers were considered as confident mutations. The consensus call sets were further reviewed for the variant allele frequency, supporting read depth, mapping quality, and strand bias to remove additional artifacts. The variant annotation was performed by Annovar^25^.

### Mutation detection of tumor-only FFPE WGS samples

Paired-end reads were mapped against human genome GRCh38 by BWA. Variants were called by Mutect2 using the tumor-only calling mode. The FFPE artifacts due to formaldehyde deamination of cytosines (C > T) were filtered via the GATK FilterByOrientationBias tool^26^. A panel of normal (PON) was constructed using 15 tonsil FFPE samples and compared with the call-sets to exclude FFPE artifacts. Multiple filtering steps were applied to exclude potential calling artifacts. The variants passing the filtering steps fulfilled the following criteria: coverage depth of the variant > 10, variant allele frequency > 0.02, alternative allele count >  = 4, allele population frequency in public databases < 0.01 (gnomadAD, 1000 genomes, ExAC and Exome Sequencing Projects), mappability > 0.7, not co-localized with repeat elements and GC percentage between 0.4 and 0.6. The variant annotation was performed using Annovar.

### Mutation detection of tumor-only FFPE RNA samples

The adapters in sequencing reads were trimmed with “trim_galore” (v0.4.4, https://www.bioinformatics.babraham.ac.uk/projects/trim_galore/, -q 20 –phred 33 –paired) and were mapped using STAR^27^. The GATK SplitNCigarReads tool was used for adjusting the cigar string of RNAseq BAMs, and the resulting BAMs were run through BQSR to calibrate base quality. The variant was called by GATK HaplotypeCaller and filtered by the VariantFiltration tool (-window 35 -cluster 3 –filter-name FS -filter "FS > 30.0" –filter-name QD -filter "QD < 2.0"). Further filtering of the variants was performed using the same criteria as described in the tumor-only FFPE calling. The variant annotation was performed using Annovar.

### Fluorescent in-situ hybridization studies

Dual-color FISH was performed on 4 µm paraffin-embedded tissue sections (Abbott Molecular: FOXO1 (Centromeric) SpectrumGreen Cat# 05J48-014; FOXO1 (Telomeric) SpectrumOrange Cat# 05J48-013). Probes were co-denatured with the target cells on a slide moat at 90 °C for 12 min. The slides were incubated overnight at 37 °C on a slide moat and then washed in 4 M Urea/2xSSC at 25 °C for 1 min. Nuclei were counterstained with DAPI (200 ng/ml) (Vector Labs) for viewing on an Olympus BX51 fluorescence microscope equipped with a 100-W mercury lamp; FITC, Rhodamine, and DAPI filters; 100X PlanApo (1.40) oil objective; and a Jai CV digital camera. Images were captured and processed using the Cytovision software from Leica Biosystems (Richmond, IL).

## Results

### Description of Cohort

Patients were selected from the anatomic pathology archives based on sample availability for histopathologic review and methylome analysis. Histopathologic and clinical data were tabulated from the electronic medical record (Supplemental Table 1). The remaining “pediatric” cohort included samples from patients treated in a pediatric hospital setting, with a few patients representing young adults. This cohort included samples from 70 female and 76 male patients. Age ranged from 3 months to 27 years (mean 7.8 years, median 7 years). Initial histologic diagnosis included: ARMS (n = 53), ERMS (n = 46), SC/SRMS (n = 3), RMS not otherwise specified (n = 44). Cases designated as NOS were enriched by our consultation practice, and included cases for which a definitive diagnosis was not reached in the initial clinical workup. This occurred secondary to poor sample quality, inability to perform ancillary studies, and in the case of inconclusive histologic findings.

The adult pleomorphic rhabdomyosarcoma subset (n = 8) included 4 female and 4 male patients. Age ranged from 61 to 79 years (mean 69.8 years, median 68.5 years).

### DNA methylation profiling of rhabdomyosarcoma

**Figure 1:**
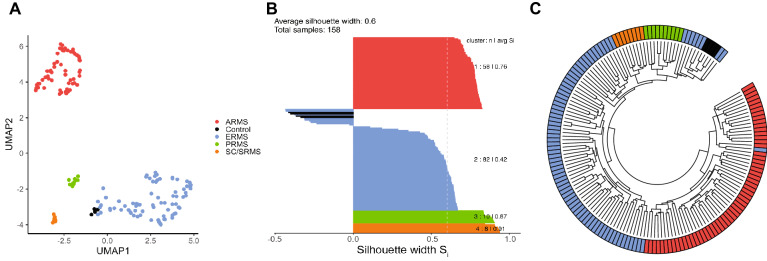
Unsupervised Clustering Analysis of 158 samples to identify potential molecular subtypes. (**A**) UMAP dimension reduction of 158 samples using the 5 statistically significant principal components determined from the 10,000 most variably methylated probes, as measured by the standard deviation of the probe-level beta values across samples. The analysis yielded 4 distinct clusters, representing 4 molecular subtypes (**B**) Silhouette analysis showing the average silhouette widths of the 4 molecular groups identified by DBSCAN based on Euclidian distances among 158 samples on UMAP coordinates. Red—ARMS, blue—ERMS, green—PRMS, and orange—SC/SRMS, black: controls (which were clustered with ERMS subtype). (**C**) Hierarchical clustering of the St. Jude cohort.

To determine if their genome-wide methylation signature could separate the types of rhabdomyosarcoma, we performed Infinium EPIC 850 K methylation array testing on a cohort of 154 rhabdomyosarcomas and four controls (skeletal muscle samples), followed by cluster analysis. Four main clusters emerged, which we designated ARMS, ERMS, SC/SRMS, and PRMS based on the most dominant histopathologic diagnosis represented in each cluster (Fig. 1). Normal control tissue grouped closely with ERMS, a finding which has been observed previously^12,28^.

### Copy number analysis in subtypes of rhabdomyosarcoma

**Figure 2:**
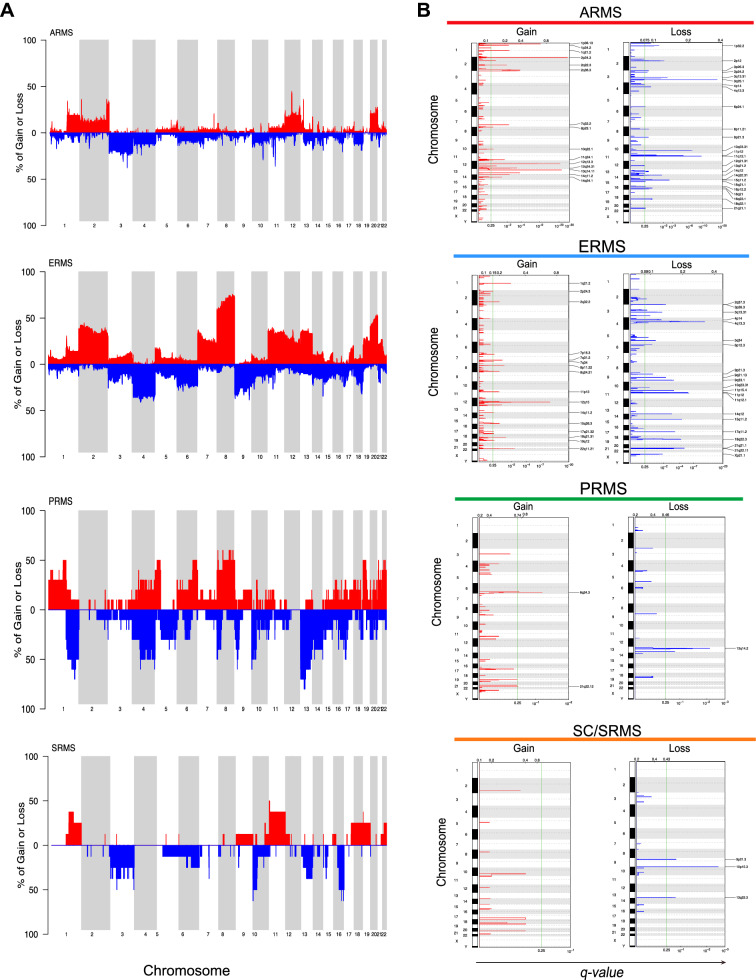
Chromosomal copy number variations among RMS molecular groups compared to reference tumors. (**A**) Copy number frequency plots of ARMS, ERMS, PRMS, and SC/SRMS molecular groups were constructed by *conumee* R package using copy number profiles of reference tumors at 0.18 threshold. (**B**) Copy number gains and losses in each molecular group determined by GISTIC 2.0. Green line indicates the *q*-value threshold to be considered statistically significant. Red: chromosomal gain, blue: chromosomal loss.

We evaluated for recurrent copy number abnormalities, focal or chromosome level, in the RMS molecular groups using the methylation array data. While few recurrent broad copy number abnormalities were detected in alveolar rhabdomyosarcoma, the ERMS group demonstrated typical changes previously reported to be associated with that subtype, including enrichment for gains of chromosomes 2, 8, 11, 12, 13, and 20 (Fig. 2A) and most of these gains were found to be statistically significant by GISTIC2.0 (Supplemental Table 2)^29^. Focal changes in ARMS included a gain of 12q13 and 13q14, containing *MYO1A*, *STAT6*, and *FOXO1* genes, while the ERMS group had a gain of 12q15, containing both the *FRS2* and *MDM2* genes. The molecularly defined PRMS subtype demonstrated complex copy number changes with a frequent gain of 1p (Supplemental Table 2). Other abnormalities included loss of 13, including focal changes at the *RB1* locus. Among focal changes, the PRMS group was also characterized by frequent gain of 6q24.3, a region containing *FBXO30*, an F-box gene known to show myocyte-specific expression (Fig. 2). The SC/SRMS group was characterized by frequent broad gain of chromosomes 11 and 22q, and loss of 10p, 13q, and 16q (Supplemental Table 2). Focal deletion in 9p21.3 encompassing the *CDKN2A* locus was also identified in the SC/SRMS group (Fig. 2B).

### Next-generation sequencing and fluorescence in situ hybridization of rhabdomyosarcomas

**Figure 3:**
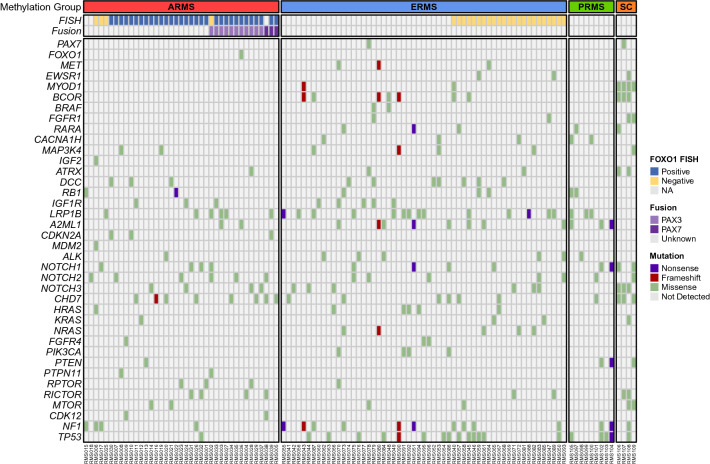
Mutation detection among RMS molecular groups using WGS and RNA-seq. Next generation sequencing was performed on tumors with sufficient material from the methylation cohort. Gene mutations (violet—nonsense, dark red—frameshift, green—missense, light gray—not detected) with VAF ≥ 20% are shown along with FISH results for FOXO1 fusion status (dark blue—positive, gold—negative, light gray – unknown) and PCR results (light purple—PAX3-FOXO1 fusion positive, dark purple—PAX7-FOXO1 fusion positive, light gray—unknown) in each RMS sample. The heatmap was split into ARRMS (red), ERMS (blue), PRMS (green), and SC/SRMS (orange).

We performed next-generation sequencing of 109 RMS (57 ERMS, 39 ARMS, 9 PRMS, 4 SC/SRMS) tumors with sufficient material that had undergone methylation analysis (Fig. 3). The tumors in the ARMS methylation class were characterized by a high proportion of *PAX3/7*-*FOXO1* fusions (49/52, 94%), and *FOXO1* fusions were not found outside the ARMS methylation group. In contrast, RMS samples in the ERMS group were enriched for mutations in activators of the RAS and AKT pathways, including *NRAS*, *HRAS*, *KRAS*, *PIK3CA*, and *NF1.* Of the two novel molecular groups, those falling in the SC/SRMS demonstrated *MYDO1* L122R mutations in all samples (n = 8, 4 via DNA comprehensive sequencing, 4 manually extracted from RNAseq data). This group also showed an increased incidence of *FGFR1* mutations (in 50% of cases, vs. 0% in ARMS and PRMS, 2.4% in ERMS). Mutations in *ATRX* and *BCOR* were proportionally higher within the SC/SRMS molecular group than in the other molecular groups. The two ERMS with *MYOD1* alterations did not harbor the L122R variant (both showed p.R121C, considered to have uncertain significance). The PRMS molecular group was devoid of recurrent activating mutations but did have a high frequency of inactivating mutations in tumor suppressors, including *TP53*, *RB1*, *NF1*, and *PTEN* Similar molecular alterations were identified in the histologically classified pediatric ERMS cases that clustered with the PRMS methylation group, including mutations in *RB1* and *TP53*. No cases of rhabdomyosarcoma with *VGLL2*, *NCOA2*, or *CITED* fusions were identified.

Break-apart FOXO1 fluorescence in situ hybridization (FISH) was attempted in all cases with sufficient materials (n = 88) and was successful in 85 cases (97%). A positive rearrangement was identified in 49 of 52 ARMS and zero cases of ERMS (0/32).

### Histologic correlates of PRMS and SC/SRMS

**Figure 4:**
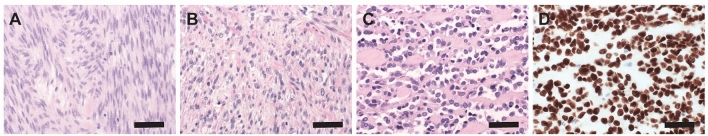
Histology of tumors in SCRMS with MYOD-1 alteration methylation group. The dominant phenotype consisted of bland spindle cells (**A**,**B**) with hyalinized stroma (**C**). When immunohistochemistry was available for MYOD1, the tumors were characterized by strong, diffuse immunoreactivity (**D**). Scale bars represent 40 µm.

Clinicopathologic evaluation of the RMS samples was performed both by chart review and microscopic evaluation, emphasizing the histology of the novel molecular groups. The methylation-defined ARMS and ERMS were concordant with the corresponding histopathologic designation in most instances (51 of 53 ARMS; 96%) and (45 of 46 ERMS; 98%). The SC/SRMS with MYOD1 abnormality were designated as spindle cell RMS in a subset of initial cases (2 of 3 initially diagnosed as SC/SRMS were truly MYOD1 mutant; 67%), though most were designated as RMS not otherwise specified (NOS) clinically (n = 6). The tumors, in most instances, were dominated by a spindle-cell morphology (Fig. 4A,B), though in some examples, the characteristic hyalinized stroma was the dominant phenotype (Fig. 4C). For those SC/SRMS-MYOD1 mutant tumors that were available for immunostaining, we observed strong and diffuse immunoreactivity for MYOD1 (Fig. 4D).

**Figure 5:**
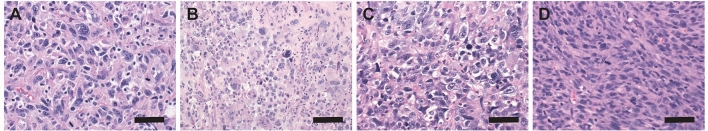
Histology of tumors in the PRMS methylation group. Tumors from adult patients (**A**,**B**) and pediatric patients (**C**,**D**) were similar in appearance. PRMS tumors demonstrated severe anaplasia with large, atypical cells, frequent mitotic activity, and tumor giant cells. Scale bars represent 40 µm.

While the PRMS methylation group predominately consisted of cases corresponding to the adult-type PRMS histologic class, a small proportion of pediatric patients clustered in that molecular group (n = 3). These pediatric tumors in this methylation subtype demonstrated severe morphologic anaplasia, analogous to the histologic appearance of the adult PRMS tumors (see Fig. 5).

**Figure 6:**
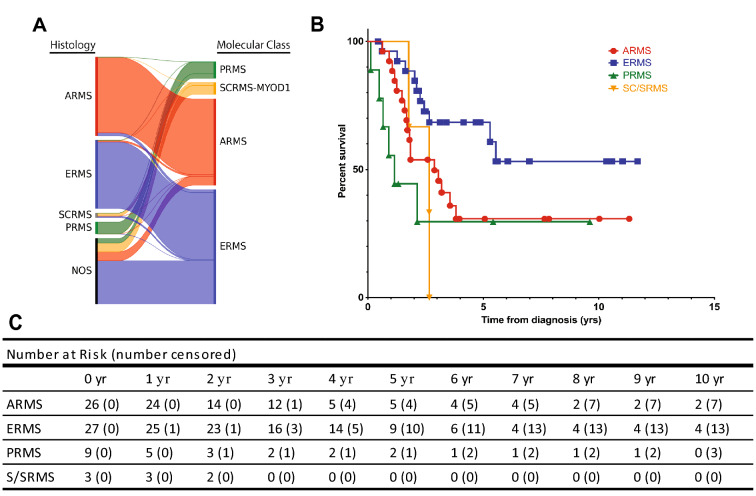
Suggested molecular re-classification of RMS and Clinical Outcome. The histologic type designated at diagnosis was compared to the molecular classification by methylation profiling and displayed as a Sankey diagram (**A**). Methylation profiling was able to classify a significant number of tumors that could not be classified using traditional histology. Clinical outcome data in the Kaplan–Meier plot (**B**) highlights poor outcome in the pleomorphic, alveolar, and spindle/sclerosing groups. Numerical data (**C**) displaying number at risk over a ten year period.

The tumors included in this study displayed enrichment for those with diagnostic ambiguity (clinically classified as RMS not otherwise specified, NOS). A molecular class was assignable in all RMS, NOS cases (n = 43, Fig. 6A) with the majority of cases assigned to the ERMS group (n = 28), and a few cases falling into ARMS (n = 6), SC/SRMS (n = 6), and PRMS groups (n = 3).

### Clinical Correlation of RMS methylation groups

The consultative nature of the cohort limited the amount of follow-up data available (Fig. 6B-C). Of the three pediatric and adolescent cases clustered with adult type-PRMS, follow-up was available for one patient who died of disease (Table 1). Similarly, clinical follow-up was available for three patients with MYOD1-mutant SCRMS, all of whom succumbed to their disease (Table 2). The adults with PRMS patients had a high mortality rate, with five of the six patients with clinical follow-up dying of disease (overall 83%).

**Table 1:** Clinical data for the pleomorphic rhabdomyosarcoma DNA methylation cluster.

Case	Age group	Age	Gender	Initial chart diagnosis	Final morphologic diagnosis	DNA Methylation Cluster	Outcome
1	Adult	61	Male	Pleomorphic Rhabdomyosarcoma	Pleomorphic Rhabdomyosarcoma	pRMS	Deceased
2	Adult	65	Male	Pleomorphic Rhabdomyosarcoma	Pleomorphic Rhabdomyosarcoma	pRMS	Deceased
3	Adult	66	Female	Pleomorphic Rhabdomyosarcoma	Pleomorphic Rhabdomyosarcoma	pRMS	Deceased
4	Adult	67	Female	Pleomorphic Rhabdomyosarcoma	Pleomorphic Rhabdomyosarcoma	pRMS	Alive
5	Adult	70	Male	Pleomorphic Rhabdomyosarcoma	Pleomorphic Rhabdomyosarcoma	pRMS	Deceased
6	Adult	74	Male	Pleomorphic Rhabdomyosarcoma	Pleomorphic Rhabdomyosarcoma	pRMS	Alive
7	Adult	76	Female	Pleomorphic Rhabdomyosarcoma	Pleomorphic Rhabdomyosarcoma	pRMS	Deceased
8	Adult	79	Female	Pleomorphic Rhabdomyosarcoma	Pleomorphic Rhabdomyosarcoma	pRMS	Alive
9	Pediatric	14	Male	Rhabdomyosarcoma NOS	Embryonal Rhabdomyosarcoma with Anaplasia	pRMS	Unavailable
10	Pediatric	16	Female	Rhabdomyosarcoma NOS	Embryonal Rhabdomyosarcoma with Anaplasia	pRMS	Unavailable
11	Pediatric	9	Male	Rhabdomyosarcoma NOS	Embryonal Rhabdomyosarcoma with Anaplasia	pRMS	Deceased

**Table 2:** Clinical data for MYOD1-mutant spindle cell/sclerosing rhabdomyosarcoma cluster.

Case	Age	Gender	Initial diagnosis	Outcome
1	2	Male	NOS, with spindled and embryonal patterns	Unavailable
2	4	Female	SC/SRMS	Deceased
3	4	Female	NOS, with spindled and embryonal patterns	Deceased
4	11	Female	NOS, with mixed spindled and alveolar patterns	Unavailable
5	15	Male	SC/SRMS	Unavailable
6	15	Female	NOS, with mixed spindled and embryonal patterns	Unavailable
7	17	Male	NOS, scant sample	Unavailable
8	21	Female	NOS, with spindled and embryonal patterns	Deceased

*NOS* not otherwise specified, *SC/SRMS* Spindle Cell/Sclerosing Rhabdomyosarcoma.

## Discussion

Rhabdomyosarcomas represent a heterogeneous group of soft tissue sarcomas associated with poor clinical outcomes. Historically, risk stratification within RMS has primarily been based on histopathologic subtype, with ARMS tumors getting more intensive therapy^30,31^. Because *FOXO1* fusions characterize most ARMS, these can be identified molecularly. Notably, only 94% of our ARMS molecular group were found to harbor a FOXO1 gene rearrangement by FISH. This observation correlates with the historically recognized fusion-negative ARMS group and demonstrates the limitations of relying on FISH alone for molecular stratification^32^.

Reliable diagnostic or prognostic biomarkers for all remaining RMS groups have not been established. ERMS are characterized by heterogeneous mutations that lead to activation of the RAS or AKT pathways but have no single recurrent mutation or fusion that can easily be tracked^32^. The most recent edition of the WHO Classification of Tumours, *Soft Tissue and Bone Tumours* now includes a histologic type encompassing the spindle cell/sclerosing subtypes of RMS, a subset of which harbor MYOD1 L122R point mutations and have particularly aggressive clinical behavior and inferior outcomes^7^. Other tumors in the SC/SCRMS group are reported to contain heterogeneous fusions, including those involving *VGLL2* or *NCOA2*, and have a less aggressive clinical course.

DNA methylation profiling has emerged as a promising method for discovering molecular heterogeneity in solid tumors, including soft tissue sarcomas^33–36^. Additionally, by combining the technology with supervised machine learning methods, neoplastic and nonneoplastic tissues can be classified using their genome-wide DNA methylation signatures in a single clinical assay. Methylation signatures correlate with cellular developmental pathways and closely recapitulate existing histologic and molecular classification schemas. Previous studies utilizing methylation profiling to interrogate RMS reported two methylation groups corresponding to the dominant ARMS and ERMS subtypes^11^. A methylation class corresponding to MYDO1-mutant SC/SRMS was recently reported as part of a comprehensive sarcoma classifier^37^. Our cohort provides independent validation of the SC/SCRMS group with MYOD1 mutation and extends those findings to include a methylation class corresponding to the adult-type pleomorphic RMS histologic groups.

Despite the reported molecular heterogeneity of SC/SRMS, we only detect a methylation group for the SC/SRMS tumors with MYOD1 L122R missense mutations. SC/SRMS with MYOD1 mutations are well-established and associated with a dismal prognosis^38^. Despite enriching for RNA sequencing in tumors histologically diagnosed as SC/SRMS, we did not find a corresponding methylation group of SC/SRMS containing gene fusions. This could suggest that SC/SRMS with fusions form a subset of ERMS or that fusion-positive SC/SRMS were represented infrequently in our cohort, with insufficient representation to form a distinct cluster. One histologically-defined SC/SRMS grouped with ERMS by methylation profiling. Unfortunately, sufficient material could not be obtained from the case to evaluate for fusions by RNA sequencing. Conceptually, this classification process is also further hindered by the knowledge that subsets of ERMS will display spindled morphology, a finding that will continue to hamper morphologic classification systems moving forward.

We also identified a second novel methylation group corresponding to the histologic adult-type PRMS group. Our PRMS methylation group demonstrated no recurrent driver mutations but was characterized by genomic instability and a high proportion of mutations in tumor suppressor genes. Although histologically-defined PRMS has primarily been considered specific to the adult population, with a peak age in the 60 s and 70 s^1^, we identified three pediatric patients in the molecularly-defined PRMS group (aged 9, 14, and 16 years). This finding challenges the notion of adult- and pediatric RMS being completely distinct diseases and suggests that a subset of pediatric ERMS with anaplasia may be molecularly indistinguishable from adult-type PRMS. In this cohort, only 3 of 13 cases classified as ERMS with anaplasia clustered with the adult-type PRMS, suggesting morphologic features alone are insufficient to identify such cases. SC/SRMS has also been found across the entire age spectrum, from infancy to adulthood, suggesting that the rare molecular groups may have a specific predilection to cross age boundaries.

Sample size limitations are often encountered in studies exploring rare malignancies. Despite the relatively low case numbers, our unsupervised analysis supports that these represents bona fide molecular groups. The number of cases for each of the rare groups (11 for PRMS and 8 for SC/SRMS, respectively) is in line with the size of molecular groups that were used to train supervised classifiers in clinical laboratories for brain tumors (19 of 91 subclasses have between 8 and 11 examples)^39^ and sarcomas (34 of 65 subclasses are limited to 8–11 examples)^37^.

Clinical outcome data were limited in our cohort, and specifically for the new novel methylation groups. The single pediatric case in the PRMS methylation subtype with follow-up experienced rapid clinical decline and died of disease 43 days following diagnosis. The tumors in the SC/RMS methylation class also were associated with poor outcomes in our cohort. Additional characterization of the clinical correlations and outcome data will be required to characterize these novel molecular groups further.

## Supplementary Information

Supplementary Information 1.

Supplementary Information 2.

Supplementary Information 3.

## Data Availability

The methylation datasets generated during the current study are available in the GEO datasets repository. Accession number GSE167059.
