# Supplementary material for: Methylation profiling reveals novel molecular classes of rhabdomyosarcoma
Source: Sci Rep. 2021 Nov 15;11:22213. doi: 10.1038/s41598-021-01649-w (PMC8592993; doi:10.1038/s41598-021-01649-w)
Supplement: Supplementary file 2 — Supplementary Information 2. [file 41598_2021_1649_MOESM2_ESM.pdf]

Arm	# Genes	Amp frequen	Amp frequen	Amp z-score	Amp q-value	Del frequency	Del frequency	Del z-score	Del q-value
1p	2121	0.03	0.04	-1.16	0.983	0.12	0.12	1.09	0.55
1q	1955	0.21	0.21	3.51	0.00123	0.03	0.04	-0.993	0.985
2p	924	0.24	0.24	4.42	4.34E-05	0	0	-1.98	0.985
2q	1556	0.26	0.26	4.83	9.99E-06	0	0	-1.98	0.985
3p	1062	0	0	-2.01	0.983	0.22	0.22	3.93	0.000471
3q	1139	0	0	-1.99	0.983	0.24	0.24	4.4	8.01E-05
4p	489	0	0	-2.1	0.983	0.14	0.14	1.58	0.271
4q	1049	0	0	-2.12	0.983	0.14	0.14	1.54	0.271
5p	270	0.14	0.14	1.6	0.186	0	0	-2.1	0.985
5q	1427	0.14	0.14	1.51	0.204	0	0	-2.13	0.985
6p	1173	0.05	0.06	-0.687	0.983	0.09	0.09	0.225	0.926
6q	839	0.05	0.06	-0.672	0.983	0.09	0.09	0.244	0.926
7p	641	0.03	0.04	-1.25	0.983	0.03	0.04	-1.25	0.985
7q	1277	0.03	0.04	-1.28	0.983	0.03	0.04	-1.28	0.985
8p	580	0.07	0.07	-0.235	0.983	0.05	0.06	-0.694	0.985
8q	859	0.07	0.07	-0.287	0.983	0.03	0.04	-1.2	0.985
9p	422	0.03	0.04	-1.21	0.983	0.05	0.05	-0.754	0.985
9q	1113	0.05	0.05	-0.786	0.983	0.03	0.04	-1.24	0.985
10p	409	0.03	0.04	-1	0.983	0.17	0.18	2.67	0.033
10q	1268	0.03	0.04	-1.03	0.983	0.17	0.18	2.6	0.0339
11p	862	0.05	0.05	-0.774	0.983	0.03	0.04	-1.23	0.985
11q	1515	0.05	0.05	-0.804	0.983	0.03	0.04	-1.26	0.985
12p	575	0.22	0.23	4.05	0.000187	0.02	0.02	-1.45	0.985
12q	1447	0.24	0.25	4.44	4.34E-05	0.02	0.02	-1.45	0.985
13q	654	0.07	0.08	-0.161	0.983	0.09	0.09	0.297	0.926
14q	1341	0.03	0.04	-1.16	0.983	0.1	0.11	0.658	0.75
15q	1355	0.03	0.04	-1.16	0.983	0.1	0.11	0.657	0.75
16p	872	0.03	0.04	-1.17	0.983	0.09	0.09	0.2	0.926
16q	702	0.03	0.04	-1.17	0.983	0.09	0.09	0.209	0.926
17p	683	0.07	0.07	-0.24	0.983	0.05	0.06	-0.699	0.985
17q	1592	0.1	0.11	0.597	0.758	0.02	0.02	-1.67	0.985
18p	143	0.02	0.02	-1.62	0.983	0.1	0.11	0.686	0.75
18q	446	0.02	0.02	-1.63	0.983	0.1	0.11	0.667	0.75
19p	995	0.1	0.1	0.589	0.758	0	0	-2.16	0.985
19q	1709	0.1	0.1	0.545	0.758	0	0	-2.18	0.985
20p	355	0.29	0.29	5.92	3.46E-08	0	0	-1.9	0.985
20q	753	0.31	0.31	6.36	4.58E-09	0	0	-1.89	0.985
21p	13	0.21	0.21	3.62	0.000928	0.02	0.02	-1.46	0.985
21q	509	0.17	0.17	2.54	0.027	0	0	-2.06	0.985
22q	921	0.16	0.16	2.09	0.0814	0.02	0.02	-1.57	0.985
Xp	834	0.05	0.06	-0.565	0.983	0.14	0.15	1.72	0.235
Xq	1312	0.07	0.08	-0.0729	0.983	0.14	0.15	1.74	0.235
Yp	109	0.09	0.19	2	0.0839	0.53	0.58	13.5	0
Yq	160	0.09	0.19	2	0.0839	0.53	0.58	13.5	0
