# Supplementary material for: Methylation profiling reveals novel molecular classes of rhabdomyosarcoma
Source: Sci Rep. 2021 Nov 15;11:22213. doi: 10.1038/s41598-021-01649-w (PMC8592993; doi:10.1038/s41598-021-01649-w)
Supplement: Supplementary file 3 — Supplementary Information 3. [file 41598_2021_1649_MOESM3_ESM.docx]

Supplementary Table 1

Clinicopathologic data

Supplementary Table 2

Statistically significant copy number changes identified in rhabdomyosarcomas by chromosomal location.
